# Supplementary material for: Research on Potential Network Markers and Signaling Pathways in Type 2 Diabetes Based on Conditional Cell-Specific Network
Source: Genes (Basel). 2022 Jun 26;13(7):1155. doi: 10.3390/genes13071155 (PMC9320152; doi:10.3390/genes13071155)
Supplement: Supplementary file 1 [file genes-13-01155-s001.zip › Supplemental File S1.pdf]

Supplementary File S1

Research on potential network markers and signaling pathways in type 2 diabetes based on conditional cell-specific network

1. Prognostic analysis of hub gene

Due to the lack of corresponding prognostic data in the study of T2D at present, the prognostic analysis of T2D can't be performed directly. It is reported that Pancreatic adenocarcinoma (PAAD) is closely related to diabetes. Diabetes is not only a risk factor for PAAD, but also one of the symptoms of PAAD. In order to explore the effect of hub genes on patients with diabetes, we used the data of PAAD, which is closely related to T2D to analyze the prognosis of T2D, to reveal the mechanism of the development of T2D from another view. We found that the overall survival rate of BIRC5, ECHDC2, NGFRAP1, PTPRS in hub genes was significantly different between the high expression group and the low expression group of the four genes ($p < 0.05$). The survival rate of the low expression group of BIRC5 was much higher than that of the low expression group, while the survival rate of the high expression group of ECHDC2, NGFRAP1, PTPRS was much higher than that of the low expression group (Figure 1). The results of prognostic analyses show that hub genes play an important role in the prognosis of pancreatic cancer.

PTPRS belongs to the receptor type IIA (R2A) subfamily of protein tyrosine phosphatases (PTPs). The R2A PTP subfamily includes PTPRF, PTPR sigma (PTPRS), and PTPR delta (PTPRD), and it has been implicated in neural development, cancer, and diabetes [1]. A second member of the R2A PTP subfamily, PTPRS, has been reported to be expressed in insulin target tissues, such as liver, adipose tissue, skeletal muscle, and endothelial cells [2]. PTPRS-deficient mice exhibit lower plasma glucose and insulin levels and greater insulin sensitivity than wild-type controls, suggesting that PTPRS may affect insulin action, even if it is unclear if it is able to directly dephosphorylate the IR or indirectly modulates its activation [3].Moreover, it is reported that during the study of the changes in DNA methylation visible before the development of T2D in mice, some genes were found to have obvious altered DNA methylation. PTPRS is one of the most obvious genes, which are rich in pathways related to insulin secretion and extracellular matrix-receptor interaction, and are related to onset of T2D in the future. Of note, the majority of genes associated with diabetes incidence have not been described in GWASs for T2D. Therefore, these differentially methylated genes, including PTPRS, are unknown in previous genetic studies and are putative biomarker for early pancreatic islet abnormalities prior to T2D onset [4].

BIRC5 also known as survivin, is a member of the inhibitor of apoptosis protein (IAP) family, which counteracts apoptosis and regulates cell division [5]. High expression of survivin is associated with poor prognosis in pancreatic cancer. Therefore, survivin is an important prognostic biomarker for these tumors [6]. This corresponds to our prognosis. Ekeblad et al. reported that expression of survivin in pancreatic cancer tissues could be a useful prognostic marker in patients with this cancer [7, 8]. In summary, survivin plays an important role in PAAD, which is closely related to diabetes.

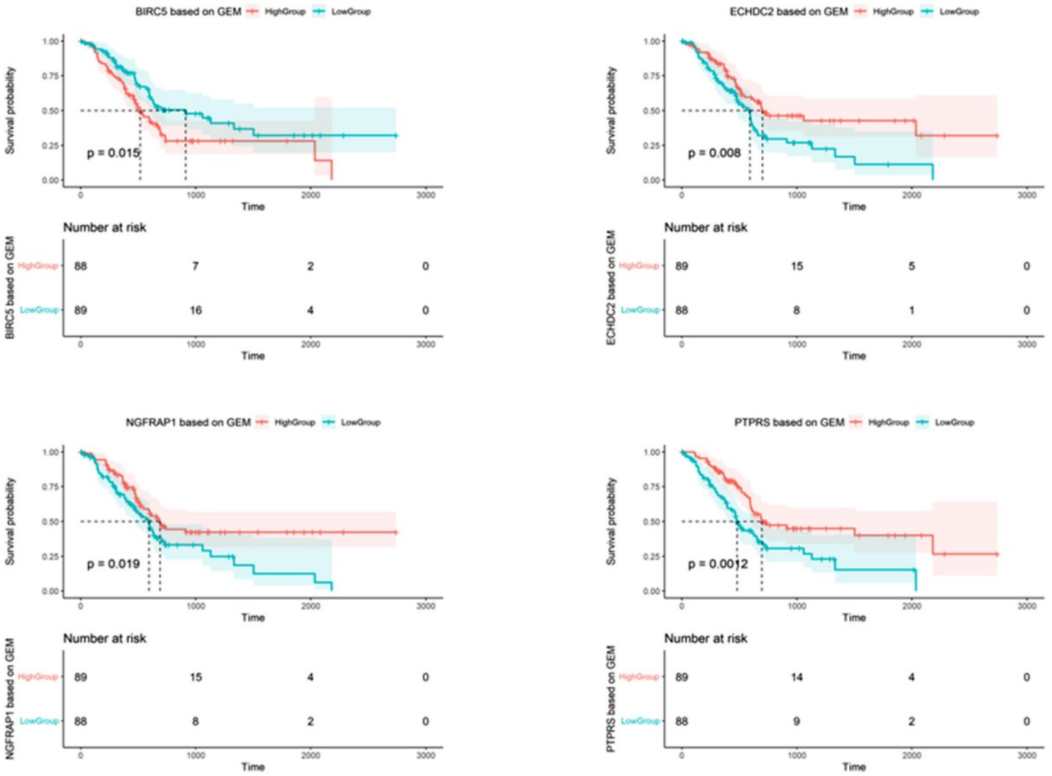

Figure S1. The prognosis curve of hub genes, the red line represents the patients with high gene expression, and the black line represents the patients with low gene expression.

2. Construction of CCSN

CCSNs provide a statistic ($\rho^{(k)}_{xy|z}$) to determine genetic associations at the single-cell level.

The general process of construction of CCSN is as follows,

[step1] Construction of Statistics

Here we define

$$\rho^{(k)}_{xy|z} = p^{(k)}_{(x,y|z)} - p^{(k)}_{(x|z)} p^{(k)}_{(y|z)} = \frac{n^{(k)}_{xyz}}{n^{(k)}_{z}} - \frac{n^{(k)}_{xz}}{n^{(k)}_{z}} \cdot \frac{n^{(k)}_{yz}}{n^{(k)}_{z}} . \qquad (1)$$

We numerically estimated the value of $\rho^{(k)}_{xy|z}$ by making a scatter diagram based on gene expression data, where $n^{(k)}_{z}$、$n^{(k)}_{xz}$ 、$n^{(k)}_{yz}$、 and $n^{(k)}_{xyz}$ are the number of cells in the neighborhood of z_k、 (x_k, z_k)、 (y_k, z_k) and (x_k, y_k, z_k) respectively.

Then, we normalize the statistic as

$$\hat{\rho}^{(k)}_{xy|z} = \frac{\rho^{(k)}_{xy|z} - \mu^{(k)}_{xy|z}}{\sigma^{(k)}_{xy|z}}, \qquad (2)$$

where $\mu^{(k)}_{xy|z}$ is the expectation and $\sigma^{(k)}_{xy|z}$ is the standard deviation. If statistic $\hat{\rho}^{(k)}_{xy|z}$ greater than N_α (N_α is the alpha quantile of the distribution), it means that gene x is related to gene y in cell k, and there is an edge, that is $w^{(k)}_{xy|z} = 1$. On the contrary, gene x and gene y are

independent in cell k, and there is no edge, that is $w_{xy|z}^{(k)} = 0$.

[step 2] The construction of CCSN for each endocrine cell

A molecular network is generally sparse, which means that a pair of genes (i.e. genes x and y) are expected to have a very small number of commonly interactive genes (as conditional genes z). The following step was used to obtain the conditional genes.

For a given cell k, we choose the top G (G≥ 1) largest importance genes as the conditional genes. We assume that the conditional gene set is $\{z_g, g = 12,3, \dots, G\}$, and the CCSN $C_{z_g}^{(k)}$ is obtained for cell k given conditional gene z_g. The CCSNs of the cell k on the condition of gene set $\{z_g, g = 1,2,3, \dots, G\}$ are $\{C_{z_1}^{(k)}, C_{z_2}^{(k)}, \dots, C_{z_G}^{(k)}\}$ Then, we use

$$\bar{C}_k = \frac{1}{G}\sum_{g=1}^{G} C_{z_g}^{(k)} = \left(C_{ij}^{(k)}\right) \qquad (3)$$

to represent the degrees of gene-gene interaction network of cell k, where $C_{ij}^{(k)}$ for $i, j = 1, \dots m$ is the (i, j) element of the matrix $\bar{C}_k$.

[step3] CNDM from CCSN

We transformed eqn. (3) to a conditional network degree matrix based on the following transformation

$$v_{ik} = \sum_{j=1}^{m} c_{ij}^{(k)} \qquad (4)$$

Therefore, for n cell networks $\{\bar{C}_1, \bar{C}_2, \dots, \bar{C}_n\}$, the CNDM which can be transformed into $m \times n$ dimension (the same dimension as the gene expression matrix) is as follows,

$$CNDM = (v_{ik}) \qquad (5)$$

where $\mathrm{i} = 1, \cdots, \mathrm{m}; \mathrm{k} = 1, \cdots, \mathrm{n}$, CNDM can reflect the direct relationship between genes from the degree of inter-action.

References

1. Chagnon, M.J.; Uetani, N.; Tremblay, M.L. Functional significance of the LAR receptor protein tyrosine phosphatase family in development and diseases. Biochem. Cell Biol. 2004, 82, 664-675. https://doi.org/10.1139/o04-120.
2. Norris, K.; Norris, F.; Kono, D.H.; Vestergaard, H.; Pedersen, O.; Theofilopoulos, A.N.; Møller, N.P. Expression of protein-tyrosine phosphatases in the major insulin target tissues. FEBS Lett. 1997, 415, 243-248. https://doi.org/10.1016/s0014-5793(97)01133-2.
3. Chagnon, M.J.; Elchebly, M.; Uetani, N.; Dombrowski, L.; Cheng, A.; Mooney, R.A.; Marette, A; Tremblay, M.L. Altered glucose homeostasis in mice lacking the receptor protein tyrosine phosphatase sigma. Can. J. Physiol. Pharmacol. 2006, 84, 755-763. https://doi.org/10.1139/y06-020.
4. Ouni, M.; Saussenthaler, S.; Eichelmann, F.; Jähnert, M.; Stadion, M.; Wittenbecher, C.; Rönn, T.; Zellner, L.; Gottmann, P.; Ling, C.; et al. Epigenetic Changes in Islets of Langerhans Preceding the Onset of Diabetes. Diabetes. 2020, 69, 2503-2517. https://doi.org/10.2337/db20-0204.
5. Altieri, D.C. The molecular basis and potential role of survivin in cancer diagnosis and therapy. Trends Mol. Med. 2001, 7, 542-547. https://doi.org/10.1016/s1471-4914(01)02243-2.
6. Xu, L.; Yu, W.; Xiao, H.; Lin, K. BIRC5 is a prognostic biomarker associated with tumor immune cell infiltration. Sci. Rep. 2021, 11, 390. https://doi.org/10.1038/s41598-020-79736-7.
7. Kami, K.; Doi, R.; Koizumi, M.; Toyoda, E.; Mori, T.; Ito, D.; Fujimoto, K.; Wada, M.; Miyatake, S.I.; Imamura, M. Survivin expression is a prognostic marker in pancreatic cancer patients. Surgery. 2004, 136, 443–448. https://doi.org/10.1016/j.surg.2004.05.023.

8. Ekeblad, S.; Lejonklou, M.H.; Stålberg, P.; Skogseid, B. Prognostic relevance of survivin in pancreatic endocrine tumors. World J. Surg. 2012, 36, 1411–1418. https://doi.org/10.1007/s00268-011-1345-7.
